# Microarray analysis of the in vitro granulomatous response to Mycobacterium tuberculosis H37Ra

**Published:** 2015-03-30

**Authors:** Niradiz Reyes, Alfonso Bettin, Ismael Reyes, Jan Geliebter

**Affiliations:** 1 Research Group of Genetics and Molecular Biology. University of Cartagena. Cartagena, Colombia; 2 Westchester Medical Center, Valhalla, NY, United States; 3 New York Medical College, Valhalla, NY, United States

**Keywords:** Mycobacterium tuberculosis, granuloma, oligonucleotide microarrays, chemokines

## Abstract

**Background:**

The hallmark of tuberculosis is the granuloma, an organized cellular accumulation playing a key role in host defense against *Mycobacterium tuberculosis*. These structures sequester and contain mycobacterial cells preventing active disease, while long term maintenance of granulomas leads to latent disease. Clear understanding on mechanisms involved in granuloma formation and maintenance is lacking.

**Objective:**

To monitor granuloma formation and to determine gene expression profiles induced during the granulomatous response to *M. tuberculosis* (H37Ra).

**Methods:**

We used a previously characterized *in vitro* human model. Cellular aggregation was followed daily with microscopy and Wright staining for 5 days. Granulomas were collected at 24 h, RNA extracted and hybridized to Affymetrix human microarrays.

**Results:**

Daily microscopic examination revealed gradual formation of granulomas in response to mycobacterial infection. Granulomatous structures persisted for 96 h, and then began to disappear.

**Conclusions:**

Microarray analysis identified genes in the innate immune response and antigen presentation pathways activated during the *in vitro* granulomatous response to live mycobacterial cells, revealing very early changes in gene expression of the human granulomatous response.

## Abstract (es)

**Antecedentes:**

La marca histológica de la tuberculosis es el granuloma, una acumulación celular organizada que cumple funciones claves en la defensa del hospedero contra *Mycobacterium tuberculosis*. Estas estructuras secuestran y confinan a las micobacterias previniendo el desarrollo de enfermedad activa; el mantenimiento a largo plazo de los granulomas conlleva al establecimiento de latencia. Un mejor entendimiento de los mecanismos involucrados en la formación y mantenimiento del granuloma es necesario.

**Objetivo:**

Monitorear la formación del granuloma y determinar los patrones de expresión génica inducidos durante la respuesta granulomatosa a *M. tuberculosis* (H37Ra).

**Métodos:**

En este estudio se empleó un modelo *in vitro *humano previamente caracterizado. La agregación celular fue examinada diariamente mediante microscopia óptica y tinción de Wright por 5 días. Para analizar la expresión génica, los granulomas fueron colectados a las 24 h, se extrajo el RNA sometiéndolo a hibridación a micromatrices de Affymetrix.

**Resultados:**

Se observó la formación gradual de granulomas en respuesta a la infección. Los granulomas persistieron por 96 h, y luego se desvanecieron.

**Conclusiones:**

Se identificaron genes de la respuesta inmune innata y vías de presentación antigénica activadas durante la respuesta granulomatosa *in vitro *a células micobacteriales vivas, lo cual reveló alteraciones tempranas de la expresión génica en el inicio de la respuesta granulomatosa humana.

## Introduction

Tuberculosis (TB) is a highly contagious disease caused by *Mycobacterium tuberculosis*, which currently threatens a significant proportion of the world population, mainly due to its ability to induce latent infection. It has been estimated that there are approximately nine million of new cases and 1.4 million deaths due to the disease each year, ranking tuberculosis second in mortality of all infectious diseases worldwide 1. Upon infection, active TB develops in 5% of individuals while 90% carry a latent infection for the rest of their lifetimes, as they are unable to fully eradicate the pathogen 2,3. During the encounter with the host, the bacilli enter the lungs in aerosolized particles infecting and activating alveolar macrophages and dendritic cells. Infected macrophages release cytokines and chemokines triggering a strong immune inflammatory response that leads to the formation of a granuloma, an organized cellular accumulation around the bacilli 4. It has been suggested that the host immune response is able to adjust and respond to the physiological state of the bacterium modulating the expression of genes directly in the site of infection 5. Complete eradication of the bacilli does not take place, since the bacterium has developed strategies not only to persist within the granuloma for long term survival, but also to exploit it for local and systemic dissemination 6. Thus, under certain physiological (malnutrition, aging, etc.) or pathological (HIV infection, diabetes, cancer, etc.) conditions, *M. tuberculosis* is able to reactivate and escape from the granuloma and disseminate 7. A better understanding of the mechanisms involved in granuloma formation and maintenance may help in the development of targeted therapies against tuberculosis 4. 

Different animal 8,9 and human *in vitro *models 10-12, have been developed to unravel the complex sequence of early molecular events involved in granuloma formation. *In vitro* models in particular represent a valuable tool for the identification of the molecular mechanisms implicated in the early immune response to defined mycobacterial cells 13. In the present study we used the *in vitro *model for granuloma development proposed and characterized by Puissegur* et al*. 11, which proved to be useful to study the molecular interactions between mycobacteria and human host cells using live mycobacteria and peripheral blood mononuclear cells (PBMCs). Using this model we determined global gene expression profiles induced during *in vitro* formation of granulomas in response to *M. tuberculosis* H37Ra strain. Analysis of genes and pathways altered during development of these *in vitro *granulomas holds the potential to aid in the understanding of early molecular events involved in this host-microbe interaction. 

## Materials and Methods

### Immune cells and bacteria

Peripheral blood was obtained from healthy donors after they signed informed consent documents. Peripheral blood mononuclear cells (PBMCs) were isolated using gradient centrifugation on Ficoll-Hipaque 1077 (Sigma Chemical Co., St Louis MO, USA) and suspended in RPMI 1640 supplemented with 10% fetal bovine serum. *M. tuberculosis* (H37Ra) cells were cultured on modified Lowenstein-Jensen Medium Base. Bacteria were collected in Middlebrook 7H9 Broth (BD Difco Biosciences, Mountain View, CA, USA) and thoroughly mixed with syringe needles. The bacteria were cultured with a serial dilution on modified Lowenstein-Jensen medium and the viability was monitored by counting the colony-forming units (CFU).

### Induction of in vitro granulomas

Peripheral blood mononuclear cells were transferred into 24 well tissue culture plates at a concentration of 1×10^5^ cells per well in RPMI 1640 with 10% FBS. Freshly prepared *M. tuberculosis* H37Ra or BCG cells were subsequently added to each well with a multiplicity of infection (MOI) of 0.1 based on trial results with different MOIs. The cells were cultured for periods from 24 h to 5 days at 37° C with medium changed every other day. To assess the specificity of the granuloma reaction, PBMCs were also cultured in the presence of *Escherichia coli* ATCC 25922 or *Staphylococcus aureus* ATCC 25923, with a MOI of 0.1. Peripheral blood mononuclear cells cultured in the absence of bacteria were also included as controls.

### Light microscopy and cell examination

To monitor the progress of cellular aggregation, cultured cells were observed under an inverted microscope (Nikon, Chiyoda-ku, Tokyo, Japan) and photographs were taken with a Nikon capture system. Cells were stained with Wright-Giemsa (W-G) modified staining (Sigma-Aldrich, St Louis, MO, USA) according to the manufacturer's instructions every 24 h up to 5 days of culture. 

### Transmission electron microscopy 

At 48 h post-infection, cellular aggregations were carefully collected, fixed for 4 h at 4^o ^C in 2% glutaraldehyde in 0.1 M cacodylate buffer with 6 mM CaCl_2, _pH 7.4. After washing with cacodylate buffer, fixed granulomas were treated for 1 h with 1% osmium tetroxide in 0.1 M cacodylate buffer, dehydrated and embedded in an Epon-araldite resin. Sections of 0.5 µm were obtained on a microtome and mounted on copper grids, stained with 3% uranyl acetate and lead citrate, and examined with a Zeiss 10 C transmission electron microscope.

### Microarray expression profiles

For microarray studies, *in vitro *granulomas were prepared in triplicates and RNA prepared at 24 h after infection of PBMCs. For control experiments, PBMCs were also cultured in triplicates at the same conditions for 24 h. Total RNAs from granulomas induced by H37Ra cells and from control PBMCs were processed at microarray facilities affiliated with New York Medical College and hybridized to individual Affymetrix Human Genome GeneChip U133 plus2 arrays (Affymetrix, Santa Clara, CA). Scanned output files were analyzed with dChip v1.3 software (www.dchip.org) and Affymetrix MicroArray Suite 5.0 (MAS 5.0). Arrays were normalized by dChip v1.3 using the invariant set normalization method 14, and model-based gene expression estimates and outlier detection algorithm were obtained according to the perfect-match-only model performed by Li-Wong 14; transcripts regarded as outliers were excluded for further analysis. Affymetrix MAS 5.0 software was used to determine if the transcripts were detected as present, absent, marginal, or no call. For significant expression level, a cut off value of 500 units was used. DNA microarray data generated from *in vitro *granulomas were compared to microarray data from uninfected PBMCs using dChip v1.3 software and the resulting expression analysis files were subjected to biological pathway and functional group analysis to determine the significance of changes at the biological context. All array data is Minimum Information about a Microarray Experiment (MIAME) compliant and the raw data has been deposited in a MIAME compliant database (GEO, Accession Number: Series GSE16250)**.**

### Pathway analysis

To identify biological pathways affected during *in vitro *granuloma formation, the microarray data were analyzed with the GenMAPP (Gene Map Annotator and Pathway Profiler) and MAPPFinder programs developed at the Gladstone Institutes at the University of California at San Francisco 15 (*www.genmapp.org*). Criteria used for GenMAPP/MAPPFinder analysis for increased expression were a minimum average intensity of 500 units, a % present (P) call of 100, and a fold change >2.0 in the *in vitro *granuloma samples compared to the uninfected PBMC samples. Criteria for decreased expression were a minimum intensity of 500 units, a % P call of 100, and a fold change of <-2.0 in the *in vitro *granuloma samples compared to uninfected PBMC samples. The programs generated a Z score based on the hyper -geometric distribution.

### Real time PCR validation of microarray data

To validate microarray expression data, quantitative real time PCR (qRT-PCR) was performed for selected genes with the same individual RNAs used in microarray experiments 16. Total RNA (1µ g) from each sample was reverse transcribed into first-strand cDNA in a 20 µL reaction volume, using QuantiTect Reverse Transcriptase kit (Qiagen) and real time quantitative PCR was performed using QuantiTect SYBR Green PCR Master Mix (Qiagen) according to the manufacturer's instructions. mRNA expression levels were assessed on the StepOne thermal cycler (Applied Biosystems, Grand Island NY, USA). Specific primers were designed for selected target genes and housekeeping genes using *Primerblast* software. Each sample was analyzed in duplicate in the PCR reaction, to estimate the reproducibility of data. 

###  Statistical analysis

All experiments were carried out in triplicate and independent experiments were also performed to assess reproducibility. Calculations of gene expression were done with Sequence Detection System 2.1 software provided by the manufacturer (Applied Biosystems) using the comparative CT method (2-^ΔΔCT)^. β-actin and Hypoxanthine-guanine phosphoribosyl transferase (HPRT) were used as housekeeping genes. Data were analyzed using SPSS 19.0 (SPSS Inc, Chicago, IL, USA). The statistical significance of changes was determined by *t*-test.

##  Results

### Infection of human PBMCs resulted in the formation of granulomas

**Figure 1.**
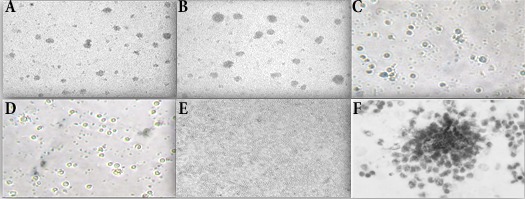
Infection of human PBMC with *Mycobacterium tuberculosis* resulted in formation of microscopic granulomas. PBMCs infected with: (A) H37Ra (100x), (B) BCG (100x), (C) *Escherichia coli* ATCC 25922 (200x), (D) *Staphylococcus aureus* ATCC 25923 (200x), or (E) uninfected PBMCs (100x). (F) Wright-Giemsa staining showing microgranulomas formed after 24 h of infection with H37Ra (400x).

**Figure 2.**
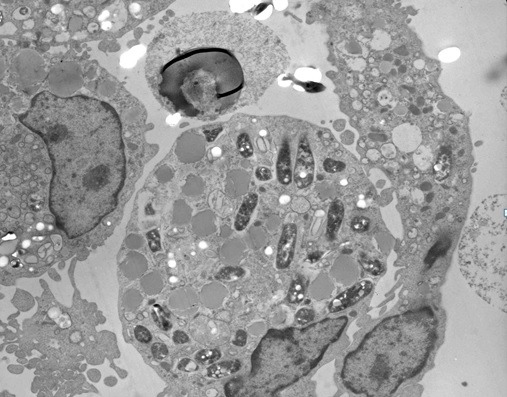
Transmission electron microscopy of H37Ra-induced granuloma. After 48 hour post-infection, cellular aggregations were collected, fixed and embedded in an Epon-araldite resin. Sections of 0.5 µm were obtained, stained and observed under transmission electron microscope (4,000x). Multiple phagosomal vesicles containing H37Ra bacteria can be observed.

To replicate granuloma formation in an *in vitro* model, we infected human PBMCs with *M. tuberculosis* H37Ra or BCG and incubated for 5 days. At 24 h of incubation, PBMCs tended to form cellular aggregations of lymphocytes in the presence of H37Ra (Fig. 1A) or BCG (Fig. 1B). Corresponding control samples from the same donors cultured in the presence of *Escherichia coli* ATCC 25922 or *Staphylococcus aureus* ATCC 25923, or cultured in the absence of bacteria did not form these aggregates (Figs 1C, 1D, 1E) indicating that cellular aggregation forms specifically in response to *M. tuberculosis* infection. The granuloma-like shape of the cell aggregates formed following 24 h of *M. tuberculosis* H37Ra infection was confirmed by Wright-Giemsa staining (Fig. 1F). Transmission electron microscopy evidenced the engulfment of mycobacterial cells by phagocytes present in the cellular aggregates at 48 h post-infection (Fig. 2). The *in vitro *granulomatous structures persisted for 96 h, and then began to disappear.

### Microarray expression profiles of in vitro granulomas induced by H37Ra

Of the total 25,690 genes analyzed with the microarrays, 2,195 were found overexpressed (fold change ≥-2, *p≤ *0.05) and 106 subexpressed (fold change *≤*2, *p≤ *0.05) in *in vitro *granulomas compared to untreated PBMCs. We found that 60% of altered genes were related to the immune response, such as antigenic processing, signaling pathways (TLR2, TNF, IL-6, IL-8, chemokines), 25% of genes were related to metabolic processes and 15% were related to oxidative stress and apoptosis (Table 1). We found overexpression of TLR2, CD14, CD86 and MyD88, which are main components of the TLR2 signaling of the innate immune response. We also found increased expression of both MHC-I and MHC-II molecules involved in antigen presentation by antigen-presenting cells (APC), a process essential to contain *M. tuberculosis* infection. In our *in vitro* granuloma model, at 24 h post-infection with *M. tuberculosis* H37Ra, we observed significant induction of a number of proinflammatory immune signaling pathways dependent on chemokines, including XCL1(3.7), XCL2(5.1), CCL2(8.3), CCL4(10.0), CCL5(5.7), CCL7(5.6), CCL8(11.1), CCL18(10.3), CCL19(2.15), CCL20(8.9), CXCL1(2.1), CXCL2(8.3), CXCL5(11.6), CXCL10(7.7), and chemokine receptors CCR2(3.8), and CXCR4(7.1). There was also increased expression of apoptosis related genes such as caspase-1 (5.0), caspase-2 (2.2), caspase-3 (3.7), caspase-4 (4.5) and caspase-9 (2.9). We also found increased expression of granulysin and granzymes A, B and H, main components of the cytoplasmic granules of cytotoxic T lymphocytes and natural killers, which are involved in cell-mediated apoptosis. We further observed increased expression of genes coding a type of peptidases involved in activation of CD8+ T cells, the cathepsins A, C, D, and W. We also identified altered expression of a number of chemokines not previously implicated in the immune response to *M. tuberculosis, *such as CCL8 (MCP-2), CCL7 (MCP-3), XCL1 (lymphotactin) and XCL2.

**Table 1.**
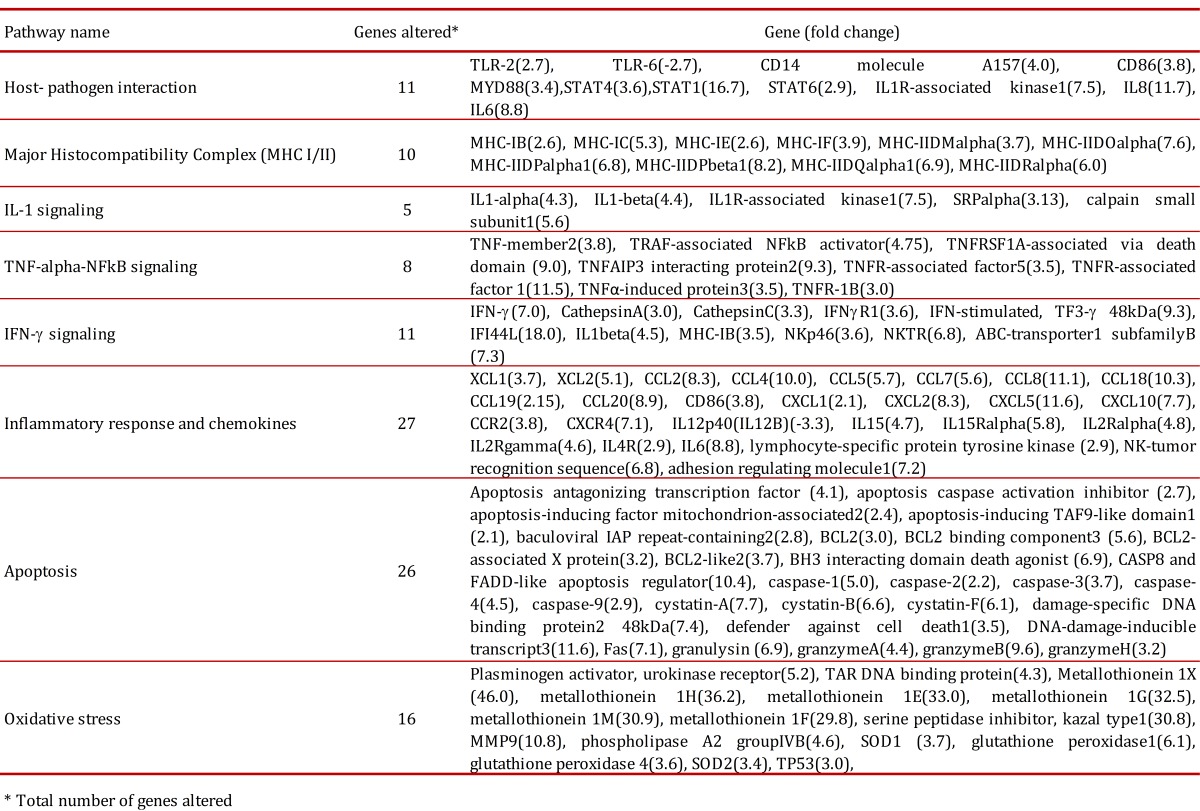
Altered pathways identified *in vitro* granulomas.

### Real time PCR validation of gene expression for selected genes

To validate microarray data, real time PCR quantitation was performed on a set of 10 genes including proinflammatory chemokines (CCL2, IL8, CCL5, CCL18), effector molecules involved in mechanisms of cell death dependent on cytotoxic T cells (cystatin B, granzyme B, granulysin), and metallothioneins (1H, 1M, 1G). Real time PCR results are presented in Table 2. Positive values indicate an increase in gene expression in *in vitro *granulomas at 24 h post-infection with H37Ra compared to uninfected PBMCs cultured for 24 h. Gene expression levels obtained by real time PCR were in agreement with corresponding levels observed in microarray experiments. 

**Table 2.**
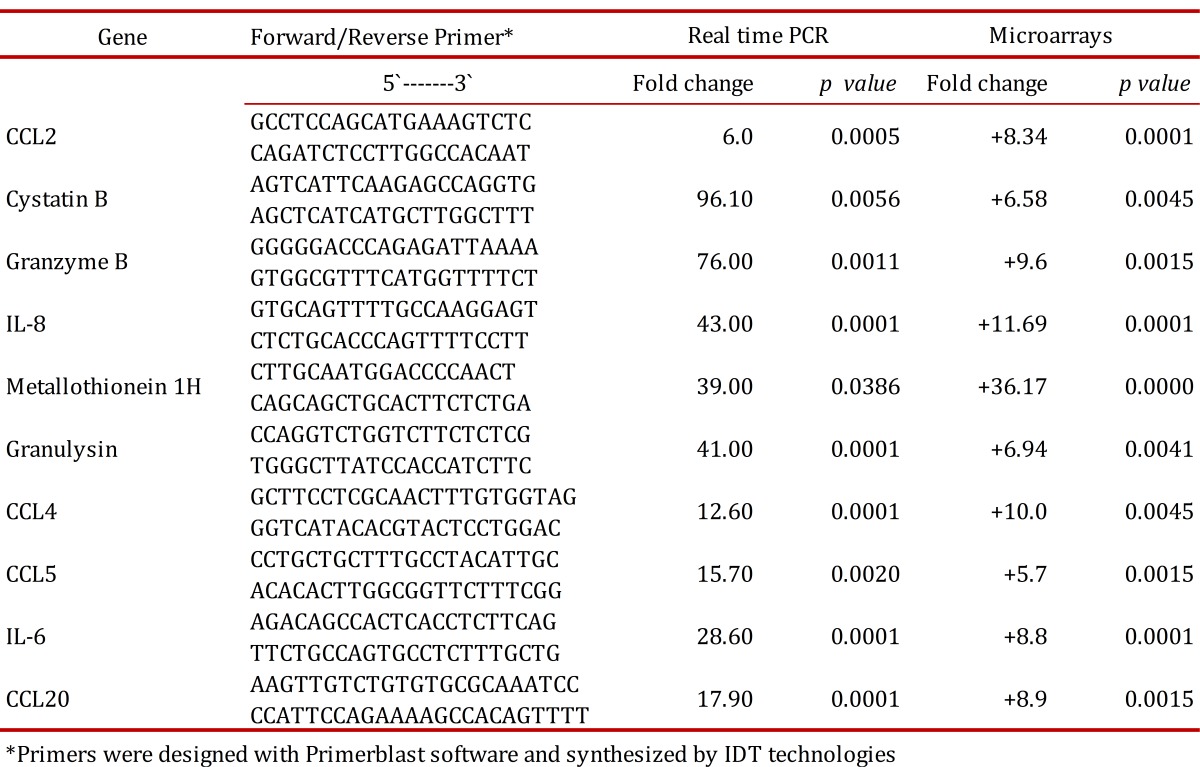
Real time PCR validation of a selected group of genes.

## Discussion

We have used a previously described model for development of *in vitro *tuberculous granulomas to determine gene expression profiles associated to this process. Using this model, we observed that lymphocytes in human PBMCs clustered around infecting bacilli resembling micro-granuloma aggregates (Fig. 1). The micro-granulomas formed specifically in response to *M. tuberculosis* infection since these aggregates did not form in response to live *E. coli* or *S. aureus* nor did they form in uninfected samples. Development and maintenance of granulomas in the lung is the main host defense against *M. tuberculosis* 17,18. Microarray analysis of these *in vitro *granulomas allowed us to gain insights into early host-pathogen interactions taking place during granuloma formation in response to *M. tuberculosis* H37Ra (ATCC 25177). Initial recognition of *M. tuberculosis* by the innate immune response involves signaling through Toll-like receptor 2 (TLR2), which is influenced by several accessory receptors, mainly CD14 17,18. Lipomannans from mycobacterial species are agonists of TLR2, that, after ligand binding, induce macrophage activation characterized by cell surface expression of CD40 and CD86, cytokine and chemokine production, antigen presentation, among other innate immune responses 19. Macrophage activation is mediated through the adaptor protein myeloid differentiation factor 88 (MyD88), but independent of either TLR4 or TLR6 recognition 20,21. Consistent with these reports, in this study we found overexpression of TLR2, CD14, CD86 and MyD88, which supports the importance of TLR2 signaling in the innate immune response to *M. tuberculosis* H37Ra. The interactions between *M. tuberculosis* and innate immune cells result in secretion of chemokines and cytokines, of which IFN-γ and TNF are particularly important in TB. One important effect of IFN-γ is to activate macrophages and enhance their expression of MHC class II molecules, leading to increased antigen presentation to T cells, an event crucial for containment of mycobacterial infection 22. Here we found that H37Ra infection resulted in increased expression of both MHC-I and MHC-II molecules involved in antigen presentation by antigen-presenting cells (APC), a process essential to contain *M. tuberculosis* infection. 

The most prominent effect observed in this study was the induction of many chemokine and cytokine genes. Several cytokines are known to play an important role in anti-TB immunity. Increased mRNA expression of IFN-γ TNF-α, IL-6, IL-8 and IL-12 in tuberculosis granuloma 23 and secretion of cytokines IL-1β, TNF-α and IL-6 in bronchoalveolar lavage fluid from involved sites of pulmonary TB have been reported 24. Transcriptome analysis of early granuloma lesions in the lungs of non-human primates exhibiting active TB, revealed a highly proinflammatory environment, expressing high levels of immune signaling pathways involving IFN-γ, TNF-α, JAK, STAT and CC/CXC chemokines 25. In our *in vitro* granuloma model, at 24 h post-infection with *M. tuberculosis* H37Ra, we observed significant induction of a number of proinflammatory immune signaling pathways dependent on chemokines, IFN-γ and TNF. 

It has been widely shown that chemokines participate in the protective immune response to *M. tuberculosis *infection 26 and many of them are chemo-attractant for leucocytes to the site of infection. Our microarray analysis revealed significant differences in the expression of CXCL- and CCL- chemokines, such as CXCL1 (Gro-α), CXCL2 (Gro-β), CXCL5 (ENA-78), CXCL8 (IL-8) and CXCL10 (IP-10). Among them, CXCL5 transcripts were the most highly over-expressed. This chemokine, along with IL-8, is induced in response to proinflammatory cytokines such as IL-1 or TNF-α, which are potent neutrophil activators 27. Previous studies have shown that CXCL5 leads to an increase in the number of infiltrating neutrophils in bronchoalveolar carcinomas and infection by* Rhinovirus* 28; however, its role in tuberculosis infection is unclear 29. 

We identified increased level of transcripts for CCL19 and CCL20 in our study. These two chemokines participate in the recruitment and activation of lymphocytes to sites of inflammation. The induction of numerous chemokines in early *in vitro *granuloma is an expected finding, since chemokine gradients are known to guide different immune effector cells to the site of infection. Chemokines have been demonstrated to be associated with mycobacterial infections, with appropriate levels of them required for preventing cells from migrating out of the granuloma, contributing in this way to maintain granuloma structure 30. 

The role played by proinflammatory immune signaling pathways involving IFN-γ and TNF-α in protection against *M. tuberculosis* infection is well understood 31. Among the overexpressed genes found in this study we found IL15 and IL15R. Several studies have provided evidence that this cytokine pathway can enhance protective immune responses against *M. tuberculosis* infection 32. Other chemokine highly over-expressed in our *in vitro* granulomas was CXCL8 (IL-8). This chemokine is implicated in the formation of tuberculous granulomas and in immunity to *M. tuberculosis* 33.

Our study identified altered expression of different apoptosis related genes, including caspases, granulysin, granzymes, and cathepsins. It has been reported that *M. tuberculosis* infection causes apoptosis of neutrophils 34 and monocytes/macrophages 34,35. Macrophages are the primary host cells for *M. tuberculosis* and consequently, there is extensive cell death among this population of cells. Specifically, it has been shown that attenuated or avirulent strains of mycobacteria, such as BCG and H37Ra, primarily induce macrophage apoptosis, while virulent strains mainly induce necrosis. These observations have generated interest in the field of TB vaccine development since pro-apoptotic mycobacterial strains able to induce greater macrophage apoptosis may stimulate a quantitatively better T-cell response 36. Cathepsin C, found overexpressed in this study, participates in activation of granzymes and in the cell death processes mediated by cytolytic T cells 37,38, whereas cathepsin W associates with NK cells, thus playing an essential role in cytotoxicity 39. The increased expression of all these pro-apoptotic genes may reflect the attempt of the innate immune system to limit the infection by restricting the growth of *M. tuberculosis*, and reveals the role played by the various caspases, granzymes and cathepsins in this process. 

There was also induction of metallothioneins and metalloproteases in the early granulomas analyzed in this study. Metallothioneins are low-molecular weight (6 to 8 KDa), cysteine-rich metal binding proteins which are induced by different stimuli, including oxidative stress, such as that generated during the respiratory burst by phagocytes 40. Metallothionein 1H has been implicated in induction of chemokines *in vitro* 41. There was a significant increase in the transcript levels of MMP9 and several cystatins. MMP9 is stabilized and protected by members of the cystatin superfamily without affecting its activity 42. Recent studies have shown the importance of MMP9 in interacting with *M. tuberculosis* secreted proteins and inducing the formation of granuloma lesions 43. It has been reported that mycobacterial lipomannan induces the expression of MMP9 in human macrophages through a mechanism dependent on TLR2 and CD14 44. The importance of Th1 cells in tuberculosis control is widely accepted 45, and our study clearly shows that *M. tuberculosis* H37Ra induces a strong Th1 response, reflected by the increased expression of Th1 cytokines such as IFN-γ, TNF-α, IL-6 and IL-1, that mediates successful resistance to *M. tuberculosis* infection 45,46. Our study supports this notion as we observed that many IFN-γ-inducible and regulated genes, such as IFI-16, IFI-30, IFI-44, IRF-3, IRF-7 y IRF-1 were increased. 

Finally, in this study we identified altered expression of a number of chemokines that have not been previously implicated in the immune response to *M. tuberculosis, *such as CCL8 (MCP-2), CCL7 (MCP-3), XCL1 (lymphotactin) and XCL2. The potential role played by these chemokines in the establishment of the tuberculous granuloma warrants further investigation. All the changes described here took place in only 24 h of infection of PBMCs by H37Ra *M. tuberculosis* strain, leading to early granuloma formation. Thus, this *in vitro* granuloma model using human PBMC is suitable for studying very early changes in gene expression taking place during induction of granuloma formation in response to *M. tuberculosis* infection. The model may be used to determine changes associated to different types of *M. tuberculosis* strains, including clinical isolates. It may also help in vaccine strategies, to decide which strains may be better candidates, based on the specific profiles of gene expression that they are able to induce.

## References

[1] WHO (2012). Global Tuberculosis.

[2] Parrish NM, Dick JD, Bishai WR (1998). Mechanisms of latency in Mycobacterium tuberculosis. Trends Microbiol.

[3] Glickman MS, Jacobs WR (2001). Microbial pathogenesis of Mycobacterium tuberculosis: dawn of a discipline. Cell.

[4] Ramakrishnan L (2012). Revisiting the role of the granuloma in tuberculosis. Nat Rev Immunol.

[5] Zhu G, Xiao H, Mohan VP, Tsen F, Salgame P, and Chan J (2003). Gene expression in the tuberculous granuloma: analysis by laser capture microdissection and real-time PCR. Cell Microbiol.

[6] Davis JM, Ramakrishnan L (2009). The role of the granuloma in expansion and dissemination of early tuberculous infection. Cell.

[7] Guirado E, Schlesinger LS (2013). Modeling the Mycobacterium tuberculosis Granuloma - the Critical Battlefield in Host Immunity and Disease. Front Immunol.

[8] Kunkel SL, Lukacs NW, Strieter RM, Chensue SW (1998). Animal models of granulomatous inflammation. Semin Respir Infect.

[9] Davis JMC, H (2002). Lewis, J. L. Ghori, N. Herbomel, P. Ramakrishnan, L. Real-time visualization of mycobacterium-macrophage interactions leading to initiation of granuloma formation in zebrafish embryos. Immunity.

[10] Kapoor N, Pawar S, Sirakova TD, Deb C, Warren WL, Kolattukudy PE (2013). Human granuloma in vitro model, for TB dormancy and resuscitation. PLoS One.

[11] Puissegur MP, Botanch C, Duteyrat JL, Delsol G, Caratero C, Altare F (2004). An in vitro dual model of mycobacterial granulomas to investigate the molecular interactions between mycobacteria and human host cells. Cell Microbiol.

[12] Birkness KA, Guarner J, Sable SB, Tripp RA, Kellar KL, Bartlett J (2007). An in vitro model of the leukocyte interactions associated with granuloma formation in Mycobacterium tuberculosis infection. Immunol Cell Biol.

[13] Rivero-Lezcano OM (2012). In vitro infection of human cells with Mycobacterium tuberculosis. Tuberculosis (Edinb).

[14] Li C, Hung Wong W (2001). Model-based analysis of oligonucleotide arrays: model validation, design issues and standard error application. Genome Biol.

[15] Dahlquist KD, Salomonis N, Vranizan K, Lawlor SC, Conklin BR (2002). GenMAPP, a new tool for viewing and analyzing microarray data on biological pathways. Nat Genet.

[16] Rajeevan MS, Ranamukhaarachchi DG, Vernon SD, Unger ER (2001). Use of real-time quantitative PCR to validate the results of cDNA array and differential display PCR technologies. Methods.

[17] Russell DG (2001). Mycobacterium tuberculosis: here today, and here tomorrow. Nat Rev Mol Cell Biol.

[18] Russell DG (2007). Who puts the tubercle in tuberculosis. Nat Rev Microbiol.

[19] Drage MG, Pecora ND, Hise AG, Febbraio M, Silverstein RL, Golenbock DT (2009). TLR2 and its co-receptors determine responses of macrophages and dendritic cells to lipoproteins of Mycobacterium tuberculosis. Cell Immunol.

[20] Vignal C, Guerardel Y, Kremer L, Masson M, Legrand D, Mazurier J (2003). Lipomannans, but not lipoarabinomannans, purified from Mycobacterium chelonae and Mycobacterium kansasii induce TNF-alpha and IL-8 secretion by a CD14-toll-like receptor 2-dependent mechanism. J Immunol.

[21] Quesniaux VJ, Nicolle DM, Torres D, Kremer L, Guerardel Y, Nigou J (2004). Toll-like receptor 2 (TLR2)-dependent-positive and TLR2-independent-negative regulation of proinflammatory cytokines by mycobacterial lipomannans. J Immunol.

[22] Harding CV, Boom WH (2010). Regulation of antigen presentation by Mycobacterium tuberculosis: a role for Toll-like receptors. Nat Rev Microbiol.

[23] Bergeron A, Bonay M, Kambouchner M, Lecossier D, Riquet M, Soler P (1997). Cytokine patterns in tuberculous and sarcoid granulomas: correlations with histopathologic features of the granulomatous response. J Immunol.

[24] Law K, Weiden M, Harkin T, Tchou-Wong K, Chi C, Rom WN (1996). Increased release of interleukin-1 beta, interleukin-6, and tumor necrosis factor-alpha by bronchoalveolar cells lavaged from involved sites in pulmonary tuberculosis. Am J Respir Crit Care Med.

[25] Mehra S, Pahar B, Dutta NK, Conerly CN, Philippi-Falkenstein K, Alvarez X (2010). Transcriptional reprogramming in nonhuman primate (Rhesus macaque) tuberculosis granulomas. PLoS One.

[26] Jo EK, Park JK, Dockrell HM (2003). Dynamics of cytokine generation in patients with active pulmonary tuberculosis. Curr Opin Infect Dis.

[27] Sachse F, Ahlers F, Stoll W, Rudack C (2005). Neutrophil chemokines in epithelial inflammatory processes of human tonsils. Clin Exp Immunol.

[28] Donninger H, Glashoff R, Haitchi HM, Syce JA, Ghildyal R, van Rensburg E (2003). Rhinovirus induction of the CXC chemokine epithelial-neutrophil activating peptide-78 in bronchial epithelium. J Infect Dis.

[29] Jang S, Uzelac A, Salgame P (2008). Distinct chemokine and cytokine gene expression pattern of murine dendritic cells and macrophages in response to Mycobacterium tuberculosis infection. J Leukoc Biol.

[30] Algood HM, Lin PL, Flynn JL (2005). Tumor necrosis factor and chemokine interactions in the formation and maintenance of granulomas in tuberculosis. Clin Infect Dis.

[31] Kaufmann SH (2002). Protection against tuberculosis: cytokines, T cells, and macrophages. Ann Rheum Dis.

[32] Lazarevic V, Yankura DJ, DiVito SJ, Flynn JL (2005). Induction of Mycobacterium tuberculosis-specific primary and secondary T-cell responses in interleukin-15-deficient mice. Infect Immun.

[33] O'Kane CM, Boyle JJ, Horncastle DE, Elkington PT, Friedland JS (2007). Monocyte-dependent fibroblast CXCL8 secretion occurs in tuberculosis and limits survival of mycobacteria within macrophages. J Immunol.

[34] Perskvist N, Long M, Stendahl O, Zheng L (2002). Mycobacterium tuberculosis promotes apoptosis in human neutrophils by activating caspase-3 and altering expression of Bax/Bcl-xL via an oxygen-dependent pathway. J Immunol.

[35] Placido R, Mancino G, Amendola A, Mariani F, Vendetti S, Piacentini M (1997). Apoptosis of human monocytes/macrophages in Mycobacterium tuberculosis infection. J Pathol.

[36] Behar SM, Martin CJ, Booty MG, Nishimura T, Zhao X, Gan HX (2011). Apoptosis is an innate defense function of macrophages against Mycobacterium tuberculosis. Mucosal Immunol.

[37] Lieberman J (2010). Granzyme A activates another way to die. Immunol Rev.

[38] Getachew Y, Stout-Delgado H, Miller BC, Thiele DL (2008). Granzyme C supports efficient CTL-mediated killing late in primary alloimmune responses. J Immunol.

[39] Stoeckle C, Gouttefangeas C, Hammer M, Weber E, Melms A, Tolosa E (2009). Cathepsin W expressed exclusively in CD8+ T cells and NK cells, is secreted during target cell killing but is not essential for cytotoxicity in human CTLs. Exp Hematol.

[40] Ruttkay-Nedecky B, Nejdl L, Gumulec J, Zitka O, Masarik M, Eckschlager T (2013). The role of metallothionein in oxidative stress. Int J Mol Sci.

[41] Aydemir TB, Blanchard RK, Cousins RJ (2006). Zinc supplementation of young men alters metallothionein, zinc transporter, and cytokine gene expression in leukocyte populations. Proc Natl Acad Sci USA.

[42] Ochieng J, Chaudhuri G (2010). Cystatin superfamily. J Health Care Poor Underserved.

[43] Volkman HE, Pozos TC, Zheng J, Davis JM, Rawls JF, Ramakrishnan L (2010). Tuberculous granuloma induction via interaction of a bacterial secreted protein with host epithelium. Science.

[44] Elass E, Aubry L, Masson M (2005). Mycobacterial lipomannan induces matrix metalloproteinase-9 expression in human macrophagic cells through a Toll-like receptor 1 (TLR1)/TLR2- and CD14-dependent mechanism. Infect Immun.

[45] Kaufmann SH (2007). The contribution of immunology to the rational design of novel antibacterial vaccines. Nat Rev Microbiol.

[46] Martinez AN, Mehra S, Kaushal D (2013). Role of interleukin 6 in innate immunity to Mycobacterium tuberculosis infection. J Infect Dis.

